# Constructing and Tuning Excitatory Cholinergic Synapses: The Multifaceted Functions of Nicotinic Acetylcholine Receptors in *Drosophila* Neural Development and Physiology

**DOI:** 10.3389/fncel.2021.720560

**Published:** 2021-09-28

**Authors:** Justin S. Rosenthal, Quan Yuan

**Affiliations:** National Institute of Neurological Disorders and Stroke, National Institutes of Health, Bethesda, MD, United States

**Keywords:** cholinergic neurotransmission, nicotinic acetylcholine receptor, neural development, synaptogenesis, *Drosophila*, synaptic plasticity, dendrite development

## Abstract

Nicotinic acetylcholine receptors (nAchRs) are widely distributed within the nervous system across most animal species. Besides their well-established roles in mammalian neuromuscular junctions, studies using invertebrate models have also proven fruitful in revealing the function of nAchRs in the central nervous system. During the earlier years, both *in vitro* and animal studies had helped clarify the basic molecular features of the members of the *Drosophila* nAchR gene family and illustrated their utility as targets for insecticides. Later, increasingly sophisticated techniques have illuminated how nAchRs mediate excitatory neurotransmission in the *Drosophila* brain and play an integral part in neural development and synaptic plasticity, as well as cognitive processes such as learning and memory. This review is intended to provide an updated survey of *Drosophila* nAchR subunits, focusing on their molecular diversity and unique contributions to physiology and plasticity of the fly neural circuitry. We will also highlight promising new avenues for nAchR research that will likely contribute to better understanding of central cholinergic neurotransmission in both *Drosophila* and other organisms.

## Introduction

**FIGURE 1:**
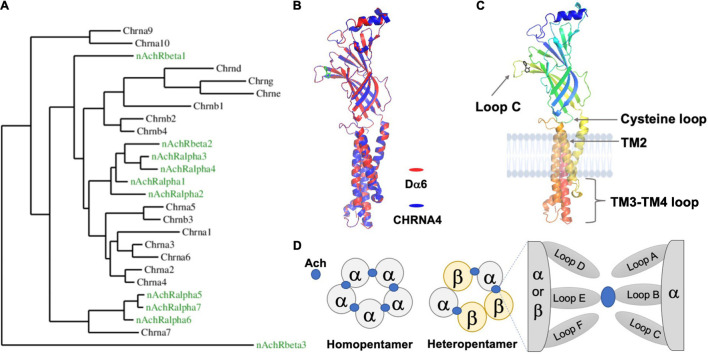
The *Drosophila* nAchR is an evolutionarily conserved ligand-gated ion channel with prototypical motifs and secondary structures. **(A)** Phylogenetic comparison between nAchR genes of *D. melanogaster* (green) and humans (black) (Taken from Rosenthal et al., 2021). **(B)** High amino acid sequence similarity between animal nAchRs permits the modeling of the *Drosophila* alpha6 (Dα6) subunit (red), using the known human alpha4 subunit (CHRNA4) X ray crystal structure (blue) as a template (Sequence comparisons were made with the Phyre2 online tool and visualized by the software PyMOL 2.5). The secondary structures and overall topology are generally conserved between the two. The ligand nicotine is labeled green. The TM3-TM4 loop for both CHRNA4 and Dα6 is discontinuous. **(C)** *Dα6* is shown in isolation and is color coded by residue position (Blue: N-terminus; Red: C-terminus). Major conserved motifs are labeled. The ligand nicotine is in black. **(D)** Schematic illustrations of the two stoichiometrically classed nAchR subtypes: homopentamers contain identical subunits whereas heteromers are composed of mixed subunits. The ligand, Ach in blue, interacts with the subunits’ interface.

One of the most ancient and frequently encountered proteins involved in nervous system communication is the nicotinic acetylcholine receptor (nAchR). nAchRs belong to the Cys-Loop Ligand-gated Ion Channel (LGIC) superfamily and form pentameric ion channels composed of five subunits, as do other members of this assemblage (Thompson et al., 2010). However, there are clear functional distinctions of nAchRs in different animal lineages. In insects, nAchRs are strictly located within the central nervous system (CNS) and are the primary means for neurons to receive fast, excitatory and inter-neuronal neurotransmission at the postsynaptic density (PSD) (Gundelfinger and Hess, 1992). Meanwhile, mammals and *C. elegans* also employ nAchRs at their neuromuscular junction (NMJ), where the receptors mediate muscle activity, and within the autonomic nervous system, where nAchRs are known to adjust sympathetic and parasympathetic tone. Notably, vertebrate nAchRs expressed in the CNS are frequently localized outside of synaptic sites and act as modulators for neurotransmitter release and neuronal excitability (Albuquerque et al., 2009; Millar and Gotti, 2009). Besides a wide range of functions, another noticeable feature of nAchRs is their molecular complexity. Even the genomes of “simpler” organisms, such as *Drosophila*, contain no fewer than ten nAchR subunit genes (Dupuis et al., 2012), which provides the basis for the enormous structural and functional diversity of the mature pentameric receptors, each with its own expression profile, channel properties and modes of regulation (Figure 1A).

Studies on acetylcholine and its receptors were founded in the vertebrate system (Langley, 1909; Changeux et al., 1970). However, once the protein sequences of all members of the *Drosophila* nAchR gene family were fully described, the powerful fly genetics system quickly produced a plethora of information, from nAchRs’ molecular architecture and cellular physiology to their participation in both simple and complex neuronal processes. Notably, while much of the initial research on *Drosophila* nAchRs evolved from a need to understand their interactions with insecticides, recent technical advances have shone light on how indispensable nAchRs are for the development and plasticity in the fly brain. Thus, studies using the *Drosophila* system have been informative both for modeling excitatory neurotransmission in insects and for probing the common roles for nAchRs at the postsynaptic specialization of CNS neurons in general. It is worth mentioning, however, due to the limited number of direct *in vivo* electrophysiological studies and structural functional analyses, there are still significant drawbacks in the fly nAchR research. For instance, to this date, there is no validated information on the native composition of nAchR pentamers in fly neurons. These long-standing limitations called for innovative approaches, which have emerged in recent years with the expansion of imaging probes, genome editing techniques and computational modeling. These new techniques greatly complement traditional *Drosophila* genetics and start to offer new insights on nAchR signaling.

Here, we provide an up-to-date description of the major aspects of *Drosophila* nAchR research accumulated over the past 40 years. While references are occasionally made to mammalian and *C. elegans* nAchRs, the reader is directed to other excellent and thorough reviews of those two systems (McGehee and Role, 1995; Dani and Bertrand, 2007; Kalamida et al., 2007; Albuquerque et al., 2009; Holden-Dye et al., 2013). We will begin with a general introduction of the molecular organization of fly nAchRs, along with their expression patterns and phylogenetics. This is followed by an analysis of the subunits’ functions, including how they react to insecticides and contribute to neural physiology at the cellular, tissue and behavioral level. The third section delineates various developmental, transcriptional, and post-translational mechanisms that regulate the expression and localization of *Drosophila* nAchR subunits. Lastly, we summarize several recent technical advances that will likely contribute to solving key outstanding questions and help us gain a better understanding of central cholinergic transmission.

## Structure, Genomics and Expression Profiles of *Drosophila* Nicotinic Acetylcholine Receptors

### Basic Features of the *Drosophila* Nicotinic Acetylcholine Receptor Subunits

The gene structure of a typical *Drosophila* nAchR subunit is similar to the prototypical nAchR gene found in other species and is characterized by several basic features, including an N-terminal extracellular domain, four transmembrane (TM) passes and a small extracellular C-terminal segment (Figures 1B,C; Sattelle et al., 2005; Dupuis et al., 2012). The major feature of the N-terminus, besides the conserved glycosylated residues and a signal peptide, is the functionally critical ligand-binding domain (LBD). According to studies in the mammalian system, only α subunits, which contain two adjacent extracellular cysteines (Cys-Cys), are capable of binding acetylcholine (Ach) through their “principal” face comprised of Loops A-C (Figure 1D; Bossy et al., 1988; Gharpure et al., 2020). The non-α subunits, β, γ, λ, and ε in vertebrates, are thought to mainly coordinate the placement of ligand within the α-subunit binding cleft *via* their “complementary” face composed of Loops D-F. By adopting this system, *Drosophila* nAchR subunits are divided into α and β groups based on the presence of the extracellular Cys-Cys motif (e.g., amino acid residues 201 and 202 in *Dα1*). However, studies suggest that some fly α-subunits do not in fact bind ligand as they lack additional key residues, similar as the case for human α5 and α10 (Albuquerque et al., 2009). This division is further complicated in *Drosophila* due to hypothesized reversions between α- and β-subunits that occurred during evolution, leading to a potential disconnection between the subunits’ nomenclature and their true ligand binding ability (Sawruk et al., 1990; Le Novere and Changeux, 1995; Dent, 2006).

The second key feature of the nAchR subunit is the group of transmembrane (TM) domains, TM1 to 4. TM2 is of particular interest, as it forms the pore-lining region cooperatively with the TM2 of the remaining four subunits, as well as the TM3-TM4 loop, which is highly variable in length between subunits, and contains predicted sites of post-translational modifications, such as phosphorylation by PKA, PKC, and PKT (Gundelfinger and Hess, 1992; Grauso et al., 2002). In the mammalian system, this loop is also involved in the assembly and synaptic clustering of the pentameric nAchR channel (Albuquerque et al., 2009; Jones et al., 2010).

### Genomics and Phylogenetics of the Fly Nicotinic Acetylcholine Receptor Gene Family

There are ten nAchR subunit genes identified in the *Drosophila* genome, of which seven are α and three are β (Littleton and Ganetzky, 2000). Despite major differences in the size of nAchR gene families across different species, there are multiple pieces of evidence suggesting a common ancestral receptor gene that likely appeared near the origin of the animal nervous system. Firstly, many phyla in the Eumetazoa utilize nAchR signaling, including chordates, nematodes, annelids, arthropods and even more basal groups such as cnidaria (Faltine-Gonzalez and Layden, 2019). Secondly, a three-gene cluster in *Drosophila*, composed of *Dα1*, *Dα2*, and *Dβ2* on Chromosome 3, is also present in mammals and includes homologs of these three fly subunits (Boulter et al., 1990; Duga et al., 2001; Chamaon et al., 2002). Finally, the primary sequence of multiple fly and mammalian nAchR genes share extensive similarities within the transmembrane domains and the extracellular region that irreversibly binds the classical nicotinic antagonist α-Bungarotoxin (α-Btx) (Bossy et al., 1988). Additionally, the intron-exon boundaries and patterns of TM3-TM4 loop glycosylation sites further revealed that some of the *Drosophila* nAchR subunits, such as *Dα1* and *Dα2*, share a closer relationship with the neuronal-specific subunits, like *CHRNA2*, present only in the vertebrate CNS, as opposed to *CHRNA1* that is restricted to the mammalian NMJ.

Beyond these basic similarities, sequence alignments have also consistently revealed the close phylogenetic relationships amongst the subunits (Sattelle et al., 2005). For instance, *Dα5*, *Dα6*, and *Dα7* form the “*α7*”-like cluster, named for the vertebrate α7 subunit which is distinct for its ability to form both homomeric and heteromeric pentamers and its high permeability to Ca^2+^ ions (Grauso et al., 2002). In contrast, *Dβ3* is identified as the outgroup, distinguished by its extremely short TM3-TM4 loop as well as the absence of an extracellular C-terminal domain (Lansdell and Millar, 2002; Dent, 2006; Dederer et al., 2011; Figure 1A).

Further nAchR phylogenetic comparisons within other insects have also revealed several intriguing observations. For example, the highly divergent subunits, such as *Dβ3* in *Drosophila*, are present in other model insects, including the mosquito *Anopheles* and the honeybee (Jones et al., 2005, 2006). Another surprising finding was that *Dα6* and its orthologs have highly conserved sites of alternative splicing and RNA A-to-I editing (Table 1; Jin et al., 2007). These changes, which were also found in *Dα4*, *Dα5*, and *Dα7*, are predicted to have functional consequences, as the edited locations often correspond to the LBD as well as multiple TM domains and their linkers (Grauso et al., 2002; Hoopengardner et al., 2003; Agrawal and Stormo, 2005; Jin et al., 2007).

**TABLE 1:** mRNA processing events associated with *Drosophila* nAchR subunits.

Gene	Alternative splicing	RNA editing
*Dα4*	Exon 2: Cassette exon in N-terminus (Lansdell and Millar, 2000a) Exon 4: Alternative exon and cassette exon in N-terminus (Cys loop) (Lansdell and Millar, 2000a)	Not Reported
*Dα5*	Exon 5: Cassette exon 5 in N-terminus (Loop F) (rare) (Grauso et al., 2002) Exons 9-11: Cassette exon in TM3-TM4 loop (Agrawal and Stormo, 2005)	Exons 10:2 events (1 amino acid altering), in TM3-TM4 loop (Grauso et al., 2002; Hoopengardner et al., 2003) Exon 14: 5 events (3 amino acid altering) in TM4/C-terminus (Grauso et al., 2002; Hoopengardner et al., 2003)
*Dα6*	Exon 3: Mutually exclusive exons between 3a and 3b in the N-terminus (Loop D of complementary subunit LBD) (Grauso et al., 2002; Jin et al., 2007) Exon 8: Mutually exclusive exons between 8a, 8b and 8c in TM2 and TM2-TM3 loop (Grauso et al., 2002; Jin et al., 2007)	Exon 5:6 events (2 are amino acid altering), in N-terminus (Grauso et al., 2002) Exon 6: 1 amino acid altering event, in N-terminus (Grauso et al., 2002) *Exon 9: 1 event (non-amino acid altering) in TM3 (Grauso et al., 2002) *Exon 10:1 event (non-amino acid altering) in TM3-TM4 loop (Grauso et al., 2002) *Exon 11: 1 event (non-amino acid altering) in TM3-TM4 loop (Grauso et al., 2002)
*Dα7*	Not Reported	Exon 10 : 1 amino acid-altering event, in TM3 (Fayyazuddin et al., 2006)
*Dβ1*	Transcripts often retain one or more introns, indicating slow or incomplete splicing (Hermans-Borgmeyer et al., 1989)	Exon 3:2 events (1 amino acid altering), in N-terminus (Hoopengardner et al., 2003) Exon 4:2 events (1 amino acid altering), in N-terminus (Hoopengardner et al., 2003)
*Dβ2*	Not Reported	Exon 9:2 events (1 amino acid altering), in TM2 (Hoopengardner et al., 2003)

Bioinformatics comparisons between genomic and transcriptomic data have identified numerous RNA processing events associated with individual nAchR subunits, either by alternative splicing and/or A to I editing. *pre-mRNA base editing generally transforms adenosine into inosine (I71). But in Dα6, cytosine may also be targeted.

### Expression, Localization and Subunit Composition of Fly Nicotinic Acetylcholine Receptors

Nicotinic acetylcholine receptors are found in many substructures of the *Drosophila* brain and ventral ganglia (Schuster et al., 1993). Early *in situ* hybridization studies on embryos revealed that transcripts of multiple subunits, such as *Dβ1* and *Dα2*, are distributed broadly in the brain and VNC (Hermans-Borgmeyer et al., 1989; Jonas et al., 1994). Promoter reporter lines, using either the 5′ UTR and/or the upstream regulatory elements, later validated these conclusions. These initial studies, although lacking cellular resolution, clearly demonstrated that the spatial distribution of the fly nAchRs are subunit-specific and developmentally controlled (Hess et al., 1994; Jonas et al., 1994).

Subunit-specific antibodies also helped determine the spatial expression patterns of nAchR genes. In general, regions positive for nAchR subunit genes overlapped well with both α-Btx binding sites and were often found in areas apposing presynaptic markers such as Acetylcholinesterase (Ace) and Choline Acetyltransferase (ChAT) (Schuster et al., 1993). Studies have shown that the medulla, lobula and lobula plate of the optic lobe are all positive for Dα1, Dα2, Dα3, Dβ1, and Dβ2 labeling, but only Dα3 staining was robustly observed in the lamina, suggesting subunit-specific functions in the adult visual circuit (Schuster et al., 1993; Jonas et al., 1994; Chamaon et al., 2000, 2002). Immunostaining also detected multiple subunits in protocerebral structures, including the mushroom body β lobes, the ellipsoid body and ventral bodies of the central complex as well as the subesophageal, thoracic and abdominal ganglia. While being informative, both *in situ* hybridization and antibody staining have limitations in their sensitivity, specificity and resolution. Recent technical advances have allowed researchers to evaluate the endogenous expression and localization of nAchRs at the single-cell level. Detailed discussions on this topic are included in the last section of the review.

One additional tool that helped characterize and isolate different nAchR subunits is affinity purification. Here, head or whole fly extract is filtered through an agarose column conjugated to nicotinic agonists or antagonists, primarily α-Btx or imidacloprid and its derivatives, and then eluted with a separate nicotinic ligand, thereby concentrating the nAchR protein. In both *Drosophila melanogaster* and *Musca domestica* samples, this affinity purification approach resulted in three distinct protein groups that range from 61 to 69 kDa (Tomizawa et al., 1996). A related technique known as photoaffinity labeling has also been used to purify nAchRs and was able to repeatedly isolate a 66 kDa-sized protein from *Drosophila* head membranes (Tomizawa et al., 1996; Tomizawa and Casida, 2003). Both of these methods were instrumental in the early stages of characterizing Dα3 and Dα5 (Chamaon et al., 2000; Wu et al., 2005). Additionally, assays on affinity-purified nAchRs uncovered discrepancies between predicted molecular weight and actual protein band size, providing experimental evidence of predicted post-translational processing, such as the glycosylation of Dα3 (Chamaon et al., 2002).

The knowledge of receptor composition is a major draw for studying the nAchRs of the mammalian brain, which is still lacking for *Drosophila* studies. The large nAchR gene family of *Drosophila* presents a significant hurdle to uncover which subunits co-assemble and in what stoichiometry. This problem has been further exacerbated by the lack of an effective heterologous system for *in vitro* expression (Ihara et al., 2020). Currently, there is still no definitive description of a functional native pentameric nAchR receptor in the *Drosophila* nervous system, although there are several lines of evidence that suggest certain subunits could co-assemble under specific experimental conditions. Early *in situ* hybridization and immunohistochemical studies consistently reported co-localization of specific subunits, which is a prerequisite, but not a proof, for co-assembly. This issue is clearly demonstrated in the case of *Dα1* and *Dβ1*: both are concentrated in the ventral bodies and lateral triangles of the central complex and within the same medulla and lobula layers of the optic lobe (Schuster et al., 1993). However, there are no direct interactions detected by co-immunoprecipitation experiments. Instead, Dα1 and Dα2 were reciprocally immunoprecipitated from adult head membrane extracts, as were Dα3 with Dβ1 (Chamaon et al., 2000; Schulz et al., 2000). Serial immunoaffinity chromatography experiments have also been conducted to support the *in vivo* association of Dα1, Dα2, and Dβ2 as a ternary complex (Chamaon et al., 2002). In general, multiple concerns have been raised due to the conflicting results generated by different biochemical methods as well as significant limitations introduced by the hybrid heterologous system. In searching for an accurate representation of native receptor interactions, the *in vitro* findings would greatly benefit from *in vivo* validation using genetic studies, which remain limited but are expanding through updated technologies, such as site-specific genome editing.

In summary, biochemical, molecular, and genetic studies demonstrate the wide distribution of *Drosophila* nAchR subunits in fly CNS, as is seen in the nervous system of many other arthropods, which is indicative of their major role in insect neurophysiology. Furthermore, genomic analyses show that the fly nAchR gene family is fairly complex and has strong homology with orthologs of other insects and even mammalian nAchRs, both at their primary sequence and predicted sites of post-translational modification. Thus, *Drosophila* nAchRs are ideal research subjects for understanding how neurons selectively use a subset of available nAchR subunits for tissue-specific functions and for modeling central cholinergic synaptic development and transmission, which will be discussed in the following sections.

## Pharmacological Properties of the *Drosophila* Nicotinic Acetylcholine Receptors

### Affinities to α-Btx and Nicotinic Analogs

Different nAchR subunits, as well as the various receptor subtypes they compose, display unique affinities and responses to common nicotinic analogs. In general, heterologous systems, such as *Drosophila* S2 cells, HEK293 cells or frog oocytes, are used to assess the total binding sites, which is a measure of surface expression, as well as the binding affinity, which indicates the strength of the ligand-receptor interaction. Furthermore, by performing membrane-solubilized vs. non-solubilized reactions with non-membrane permeable agonists, it is possible to distinguish the surface vs. internally localized receptor. These studies successfully identified subunit-specific reactions to different pharmacological treatments. For instance, the competitive agonist epibatidine interacts only with the subunits Dα1-Dα4, but not Dα5-Dα7 (Lansdell et al., 1997, 2012; Lansdell and Millar, 2000b, 2004). Differences also exist among closely related subunits: Despite strong sequence homology, the EC_50_ of nicotine for the hybrid Dα1 receptor is more than two orders of magnitude lower than that for the Dα2 hybrid (Bertrand et al., 1994; Schulz et al., 2000; Dederer et al., 2011).

The naturally occurring α-Btx, a snake venom component structurally similar to Ly6-type proteins, the endogenous inhibitors for cholinergic signaling (Wu et al., 2014), has also helped to parse out pharmacological differences. Initially used to extract and purify nAchR proteins, α-Btx has also helped define nAchR receptor biology in general. Notably, α-Btx binding is subunit-specific. *In vitro* studies using either fusion proteins containing the extracellular region, or full-length, of the nAchR subunits revealed that the *Drosophila* subunits Dα1, Dα3, Dα5, and Dα6 showed substantial affinities to α-Btx, whereas binding to subunits Dα2, Dα4 or Dα7 was negligible (Bossy et al., 1988; Bertrand et al., 1994; Lansdell and Millar, 2000a, 2004; Wu et al., 2005). In addition, α-Btx directly impairs nAchR-mediated processes in fly and therefore can be used to investigate cholinergic signaling *in vivo*, as well as understand structure/function connections for particular residues that contribute to the ligand-binding site (Pyakurel et al., 2018). It is also worth mentioning that, in general, the endogenous nAchR has a stronger affinity than the receptor reconstituted *in vitro*, possibly due to the incorrect configuration of nAchR subunits or the lack of proper post-translational processing in the *in vitro* condition (Schloss et al., 1988, 1991).

### Nicotinic Acetylcholine Receptors as Targets of Insecticides

No discussion of insect nAchRs would be complete without referencing their exploitation as pesticide targets as well as how these receptors form an evolutionary substrate for insecticide resistance. Chemical development has led to numerous classes of insecticides, which have strong adverse effects on nAchR-mediated cholinergic transmission within the insect CNS and ultimately cause lethality (Matsuda et al., 2001; Millar and Denholm, 2007). Although all of them impair nAchR activity, there are different mechanisms of action. For example, the neonicotinoids block the critical ligand-binding pocket of the receptor, whereas the spinosads are allosteric modulators. Even those belonging to the same group vary in size and charge, and thus likely display a binding preference for certain subunits with their unique 3D structures. Therefore, besides the obvious significance of these findings for the pesticide industry, this line of investigation also offers insights into the molecular distinctions and similarities among nAchR subunits and translates to better modeling of mammalian nAchRs for both research and therapeutic purposes, while minimizing negative impacts of pesticides on humans and other animals (Tomizawa and Casida, 2001; Thany et al., 2007; Millar and Harkness, 2008).

Many insects are sensitive to the neonicotinoids, a group of potent agonists with a molecular structure mirroring nicotine and thus targets the Ach-binding site in nAchR subunits. Using neonicotinoid affinity columns in combination with *Drosophila* genetic manipulations, researchers have demonstrated their differential affinity for distinct subunits. For instance, preincubation of head membranes with nicotinic ligands, such as nicotine and *d*-tubocurarine, prevents the isolation of the Dα1 subunit through the neonicotinoid affinity columns, supporting Dα1 as one of their main targets (Tomizawa et al., 1996; Tomizawa and Casida, 2003; Dederer et al., 2011). Later studies using heterologous expression systems provided additional evidence for subunit-specific insecticidal action. In particular, Dα1-Dα3 appeared to have high-affinity for various chemicals such as imidacloprid and clothianidin (Matsuda et al., 1998, 2001; Lansdell and Millar, 2000b; Ihara et al., 2004; Somers et al., 2017). In contrast, through chimeric receptor studies and direct measurement of mortality, the extracellular regions of both Dα6 and Dα7 are apparently unable to bind imidacloprid (Lansdell and Millar, 2004). Notably, this partition resembles the phylogenetic relatedness, where *Dα5, 6*, and *7* subunits are distant from the other *Drosophila* α subunits (Figure 1A).

**FIGURE 2:**
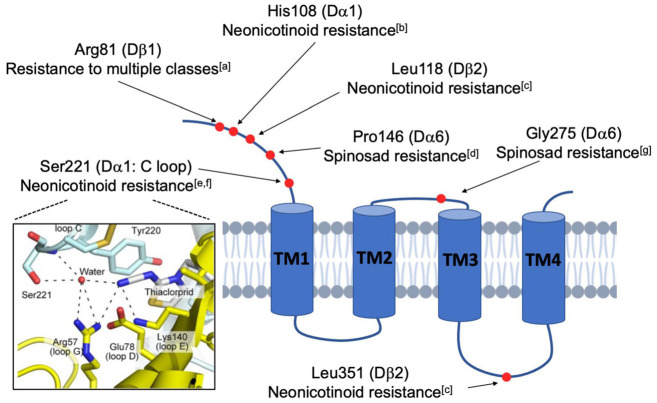
Schematic diagram illustrating the mutated residues in nAchR subunits that confer insecticide resistance. Natural and lab-derived insecticide resistance in *Drosophila* often develops from amino acid substitutions (red dots) in a single nAchR subunit. Because these resistance-endowing mutations are found at varied locations in the mature protein, it is believed that resistance occurs *via* multiple mechanisms. Some, such as the Ser221 depicted in the framed panel on the left, directly interfere with the action of competitive agonists, like the neonicotinoids, at the ligand-binding site (Image taken from Shimada et al., 2020). In contrast, others are more distal and likely indirectly impact nAchR functions by modulatory activities (^a^Homem et al., 2020; ^b^Ihara et al., 2014; ^c^Perry et al., 2008; ^d^Somers et al., 2015; ^e^Hikida et al., 2018; ^f^Shimada et al., 2020; ^g^Zimmer et al., 2016).

Another interesting case of subunit-specific sensitivity involves the tight link between *Dα6* and the activity of spinosyns. Multiple loss-of-function (LOF) alleles of *Dα6* endow the fly with high resistance to Spinosad but limited resistance to other insecticide classes, such as the avermectins and pyrethroids (Perry et al., 2007; Watson et al., 2010). Here, resistance in mutant animals is characterized by reduced mortality, elevated EC_50_ levels and a lack of Spinosyn A-induced current in the larval ventral ganglia (Perry et al., 2007, 2015; Watson et al., 2010; Rinkevich and Scott, 2013; Somers et al., 2015). Moreover, rescue experiments in a *Dα6* null background indicated that other subunits such as *Dα1*, and even the phylogenetically similar *Dα5* and *Dα7*, were unable to re-sensitize the *Dα6* mutants to Spinosad (Perry et al., 2015; Figure 2).

Beyond spinosad and *Dα6*, there are other instances of resistance resulting from site-specific mutation (Figure 2). For instance, single residue changes of *Dα1* alter neonicotinoid-induced currents of hybrid fly Dα1/Chickβ2 nAchRs *in vitro* (Hikida et al., 2018; Shimada et al., 2020). Additionally, nitenpyram resistance is linked to the cooperative activity of the subunits Dα1 and Dβ2 (Perry et al., 2008). Given that there are more examples of subunit-specific interactions with insecticides (Ihara et al., 2014; Zimmer et al., 2016; Homem et al., 2020), the general idea is that insecticide efficacy is contingent upon specific residues and motifs in distinct subunits, supporting the endeavor of developing species-specific pesticides that discriminate between the subunits of different pentamers. Another extension of these studies is the hope that computational modeling can estimate the importance of residues and structural motifs of the nAchR subunits, and provide informed predictions concerning the evolution of insecticide resistance in the field.

## Physiological Studies of Nicotinic Acetylcholine Receptors in the *Drosophila* Central Nervous System

### Nicotinic Acetylcholine Receptors as the Primary Mediators of Excitatory Synaptic Transmission in the Fly Central Nervous System

**FIGURE 3:**
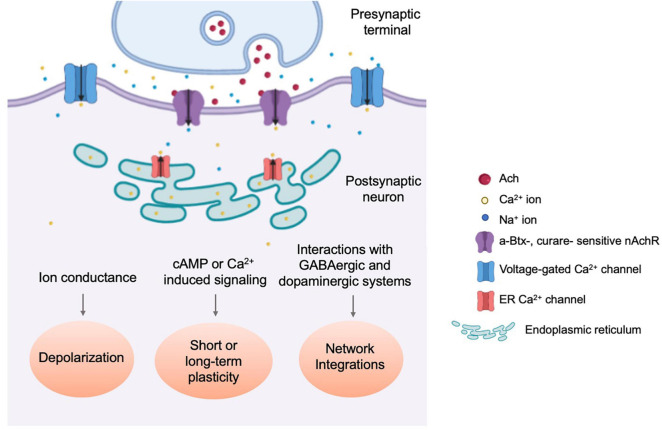
Physiological responses towards nAchR-mediated cholinergic neurotransmission. Schematic diagram illustrating the three types of physiological events which occur through nAchR-mediated cholinergic signaling. nAchR activation at the postsynaptic density results in rapid depolarization of the postsynaptic cell, increasing the probability of an action potential and signal propagation to downstream circuit components (Lee and O’Dowd, 1999). Slower, secondary changes are initiated by secold messenger systems and through integration with incoming inhibitory synaptic transmission as well as neuromodulatory input (Su and O’Dowd, 2003; Campusano et al., 2007; Kuehn and Duch, 2013).

Unlike nAchRs of the vertebrate CNS, which are mostly neuromodulatory and localize perisynaptically or extrasynaptically, fly nAchRs are likely primarily postsynaptic (Dani and Bertrand, 2007), colocalizing with postsynaptic proteins such as DLG and CamKII, while apposing presynaptic active zone molecules such as DSyd-1 (Ashraf et al., 2006; Owald et al., 2010). When a *Dα7*:GFP transgene is overexpressed in the Kenyon Cells (KC) of the Mushroom Body (MB), GFP puncta were observed within the dendritic claw-like ring surrounding Projection Neuron (PN) axon terminals marked by the active zone marker, Brp^short^:Cherry (Christiansen et al., 2011). Notably, fly nAchRs also coexist on dendrites with other neurotransmitter receptors, such as the GABA_A_ receptor *Rdl* in MN5 motoneurons, although there appears to be a spatial segregation between the two (Kuehn and Duch, 2013), supporting the potential role of dendritically localized nAchRs in generating action potentials and balancing GABAergic inhibitory input (Figure 3).

Evidence for cholinergic transmission in insects emerged in the 1970s and has since been demonstrated directly by physiological studies (Dudai and Amsterdam, 1977; Blagburn and Sattelle, 1987). *In vitro* cultured embryonic cholinergic neurons, expressing ChAT, produce fast and rapidly decaying inward currents that are readily detected as both miniature EPSCs (mEPSC) and action potential-evoked EPSCs, and can be reversibly silenced by application of the nAchR-specific antagonist curare but not GABA receptor or Glutamate receptor antagonists. Simple forms of calcium-dependent plasticity are also observed as mEPSC frequency increases following multiple rounds of KCl-induced depolarization (Lee and O’Dowd, 1999). Furthermore, nAchR-mediated cholinergic transmission was demonstrated using cultured MB neurons of dissociated pupal brains, where the majority of mEPSCs are α-Btx sensitive with a broad amplitude distribution (Su and O’Dowd, 2003; Figure 3).

There is also an extensive list of *in vivo* studies documenting the physiological importance of nAchRs. A well-studied case is the function of *Dα7* in the Giant Fiber (GF) circuit, where *Dα7* is responsible for relaying visual stimuli in the optic lobe to motoneurons in the periphery, generating a simple sensorimotor escape reflex seen in many insects. In *Dα7* null mutants, defective responses from the dorsolongitudinal muscle (DLM) are observed and result from faulty neurotransmission between lobula columnar neurons and the GF neuron as well as between the peripherally synapsing interneuron (PSI) and DLM’s motoneuron (DLMn) (Thomas and Wyman, 1984; Trimarchi and Schneiderman, 1993; Fayyazuddin et al., 2006; Mejia et al., 2010, 2013). Later studies even identified particular residues, such as a highly conserved Tyrosine 195, that are directly involved in orchestrating agonist binding (Mejia et al., 2013). Surprisingly, these obvious and robust *Dα7^–/–^* phenotypes are not observed in lobulate plate tangential neurons (LPTCs), despite the fact that these cells are also a part of the light-induced escape reflex circuit, and also clearly express postsynaptic Dα7 puncta on both the VS- and HS-type dendrites of these cells (Leiss et al., 2009; Raghu et al., 2009). The fact that only LPTC recordings appear normal in *Dα7* null animals is a strong indication that LPTC neurons, but not other cells in this circuit, experience nAchR subunit compensation. Although not fully understood, the phenomenon of one nAchR subunit substituting for another has been clearly demonstrated in mouse models as well and could provide critical information on subunits’ functional redundancies (Xu et al., 1999).

*Drosophila* nAchRs have also been investigated in the adult fly olfactory circuit, where they are active at the synapses between olfactory receptor neurons (ORN) and PNs, as well as higher order brain structures, such as the synaptic regions between the PNs and Kenyon Cells (KC) of the MBs (Kremer et al., 2010; Murmu et al., 2011). Genetic studies have even implicated specific subunits, such as *Dα5* and *Dα6* but not *Dα7*, in odor-evoked activity in the M4/6 MB Output Neurons (MBONs) (Barnstedt et al., 2016).

### The Role of Cholinergic Signaling in Neurodevelopment, Cellular Plasticity and Morphogenesis

In addition to serving as the primary means of relaying excitatory neurotransmission in the fly CNS, nAchR-mediated cholinergic signaling also functions in regulating growth and anatomical features of the dendritic arbor. In the MN5 motoneuron, activation of Dα7-containing nAchRs results in a CamKII-dependent upregulation, and the nuclear translocation, of the early activity gene AP-1, the *Drosophila* homolog of Fos/Jun. This enables the dendrite outgrowth that normally occurs during the early pupal stage. Interestingly, not only are dendrite arborizations blocked by a dominant-negative form of AP-1 transgene, but premature activity at this same synapse results in diminished branching, implicating a tight temporal control for activity-dependent dendrite development in these neurons (Vonhoff et al., 2013). Later studies further demonstrated that Dα7-mediated excitatory transmission competes with Rdl-mediated inhibitory transmission during development. While being roughly equivalent initially, GABAergic and cholinergic domains may shift when there is an imbalance of inhibitory vs. excitatory activities, respectively. This phenomenon is thought to limit the range of morphological variability in this cell type (Ryglewski et al., 2017).

Our own studies in the larval visual circuit have demonstrated *Dα6*’s functions in synaptogenesis and dendrite development. Expressed in early larval stages, *Dα6* has both autonomous and non-autonomous contributions to ventral lateral neurons’ (LNvs’) synapse formation. The loss-of-function mutants of *Dα6* show a significant reduction in both synapse number and dendrite volume (Rosenthal et al., 2021). Additionally, there has also been a report of non-vesicular Ach release that is crucial for photoreceptor targeting in the developing adult optic lobe. Transgenic disruption of cholinergic signaling *via* manipulation of a temperature-sensitive allele of *Choline acetyltransferase (Cha^ts^)* and α-Btx application resulted in abnormal axon growth cones which do not align correctly when terminating in the lamina and lead to ectopic bundles. However, the absence of these phenotypes in *VAchT* mutants and animals exposed to tetanus toxin (TTX) suggests a non-canonical mechanism of Ach release (Yang and Kunes, 2004).

Nicotinic acetylcholine receptor activity has been linked to neural plasticity in the fly CNS. In many cases, this reflects the intricate and coincident relationship between cholinergic and other types of neurotransmission (Figure 3). In cultured KCs from pupal MBs, nicotine induces calcium transients from direct Ca^2+^ ion influx through nAchRs, as well as the release from intracellular stores and voltage-gated calcium channels. These calcium transients are significantly dampened by short conditioning pulses, indicating a strong experience-dependent modification (Campusano et al., 2007). Additional experimental evidence was produced by calcium traces recorded in isolated MB neurons that are consecutively exposed to GABA and Ach. Depending on the order of treatments, the calcium responses showed changes both in peak amplitude and decay time (Raccuglia and Mueller, 2014).

The connection between nAchRs and dopamine, which is a major research subject in vertebrate models, has also been explored in *Drosophila*. Using a variety of techniques and approaches, we have learned that MB KCs and a subset of dopaminergic neurons (DAn) form reciprocal axon-axonal synapses that are critical for olfactory learning (Cervantes-Sandoval et al., 2017). In the larval VNC, stimulation of nAchRs induces dopamine release, whereas in the MB, dopaminergic input onto KC axon terminals requires simultaneous stimulation from both cholinergic PN inputs and glutamatergic inputs from the ascending VNC (Ueno et al., 2017; Pyakurel et al., 2018).

Lastly, nAchR-mediated feedback loops also play an important role in the induction of homeostatic plasticity. In the MB calyx, both pre- and post-synaptic homeostatic adaptions are observed after artificial neuronal silencing (Kremer et al., 2010; Murmu et al., 2011; Figure 3). A series of experiments using cultured cholinergic neurons have further elucidated the involvement of nAchRs in regulating neuronal homeostasis. Here, pharmacological blockade of Dα7-mediated synaptic activity upregulates Dα7 protein synthesis. In the first phase, this blockade strengthens mEPSC inward currents and lasts several hours post-inhibition. The second phase is characterized by the calcium- and CamKII-dependent potentiation of the K^+^ channel *Shal*, which reverts mEPSC frequency and amplitude toward their original, pre-stimulation values. These results demonstrated the inhibition-triggered homeostatic upregulation of synaptic activity and how it is balanced by the enhancement of the hyperpolarizing K^+^ currents (Ping and Tsunoda, 2011). These findings were replicated *in vivo* through genetic manipulation of cholinergic activity (Eadaim et al., 2020), which also implicated the role of transcription factor NFAT that acts as the intermediate linking increased Dα7-dependent synaptic transmission with *Shal* upregulation.

To summarize, fly nAchRs are essential postsynaptic ligand-gated ion channels mediating excitatory transmission across chemical synapses, and their prevalence in the *Drosophila* CNS is demonstrated by a wide range of phenotypes observed in genetic mutants and in animals exposed to nicotine-like toxins. At the cellular level, these deficiencies appear as impaired agonist-induced inward currents. On a larger scale, they can manifest as defects in olfactory and visual processing or abnormal motor reflexes. In this way, nAchRs are similar to the ionotropic glutamatergic receptors (iGluR) in the vertebrate CNS, which support excitatory transmission in the majority of central synapses, rather than the central vertebrate nAchRs, which mainly modulate neuronal excitability and presynaptic release. However, central fly nAchRs also contribute to neuronal biology beyond simply propagating action potentials. As described above, nAchR-mediated cholinergic signaling is a key driving force of dendrite morphogenesis and axon guidance and also participates in neuronal plasticity and homeostatic adaptations, the latter of which may involve integration with other modes of ionotropic or metabotropic neurotransmission (Yang and Kunes, 2004; Kremer et al., 2010; Ping and Tsunoda, 2011; Vonhoff et al., 2013; Ueno et al., 2017). In vertebrates, neuronal development and plasticity rely on iGluR-mediated neurotransmission. For example, the NMDA receptor subtype of iGluR is highly permeable to calcium and ion influx through this receptor is critical for synaptic plasticity and scaling (Malenka and Bear, 2004; Cooke and Bliss, 2006). It is also a key component in driving the maturation of glutamatergic synapses and dendrite arborization, another functional similarity between fly nAchRs and vertebrate iGluRs (Wu et al., 1996, 1999; Sin et al., 2002). The analogous relationship between these two systems has important implications for future research on *Drosophila* nAchRs. For example, although not much is known about activity-dependent post-translational modifications of *Drosophila* nAchRs, phosphorylation events of iGluRs in the vertebrate system are well characterized for their influences on both the abundance of iGluRs at the postsynaptic density (PSD) as well as their biophysical properties (Sheng and Kim, 2011; Henley and Wilkinson, 2013). This observation, in combination with the presence of multiple predicted phosphorylation sites within the TM3-TM4 intracellular loop of many fly nAchR subunits, highlights the need to understand how phosphorylation states of nAchRs impact neurotransmission in the fly CNS.

## Spatial and Temporal Regulation of the Nicotinic Acetylcholine Receptors

### Developmental Modulation of Nicotinic Acetylcholine Receptor Expression

Even during the early era of characterizing *Drosophila* nAchRs, it had become clear that the subunits and the receptors they form do not remain steady but display stark periods of up- and down-regulation. Generally, nAchR subunits’ expression is potentiated during embryogenesis, although the stage of initial detection varies by subunit (Hermans-Borgmeyer et al., 1986, 1989; Jonas et al., 1990; Schuster et al., 1993; Grauso et al., 2002). For example, the major transcript of *Dα2* is observed in 2 h old embryos, whereas *Dβ1* is not detected until late embryonic stages (Bossy et al., 1988). The end of embryogenesis usually represents the time of peak expression (Sawruk et al., 1990; Jonas et al., 1994). By the first instar larval stage, the transcript level tends to be greatly reduced and remains low through the duration of the larval stage. Expression typically rises again during the pupal and adult stages, although noticeable differences between subunits may persist (Hermans-Borgmeyer et al., 1989; Jonas et al., 1990; Sawruk et al., 1990; Grauso et al., 2002). In terms of spatial distribution, *in situ* hybridization shows strong labeling of nAchR subunits’ transcripts widely distributed in various regions of the brain and VNC, but never outside of the CNS. Antibody labeling and promoter reporter experiments produced similar spatial and temporal patterns, with the exception that nAchR protein is concentrated in the neuropil region, rather than the cortical cell body layer where the RNA signal is detected (Schuster et al., 1993; Hess et al., 1994; Jonas et al., 1994).

Anatomical studies also revealed other interesting aspects of nAchR subunit temporal regulation, such as isoform-specific expression profiles for *Dα1* during the embryonic vs. adult period (Bossy et al., 1988). Moreover, for the *Dβ1* subunit, a significant portion of RNA can be detected as incompletely spliced transcripts, and these transcripts also have a unique temporal expression profile compared to the fully spliced mRNA isoforms (Hermans-Borgmeyer et al., 1989), which may have functional consequences. Taken together, although temporal profiles of individual subunits may vary, general patterns of nAchR RNA and protein expression, which are elevated in the embryonic and pupa stages, are notably in congruence with major periods of neuronal differentiation, potentially enabling the increased production and delivery of nAchRs that contribute to cholinergic synapse development (Hermans-Borgmeyer et al., 1986; Schuster et al., 1993).

Our recent investigation on postsynaptic development in the larval LNvs has clarified the functional significance of the temporal regulation of different nAchR subunits. Early in larval development, the immature LNv expresses *Dα6* at relatively high levels, which supports synapse formation. As the synaptogenesis period ends, *Dα6* expression is suppressed while *Dα1* is upregulated to stabilize the maturing synapse and enhance neurotransmission. This instance of “receptor switching” may in fact be a general phenomenon underlying synapse maturation in the fly CNS (Rosenthal et al., 2021). Interestingly, the LNvs also display a dramatic homeostatic response toward chronic elevation of synaptic input, which leads to reduced *Dα1* expression in LNvs and dampened physiological output, suggesting that *Dα1* also acts as the activity-regulated effector mediating the LNv’s homeostatic response.

**FIGURE 4:**
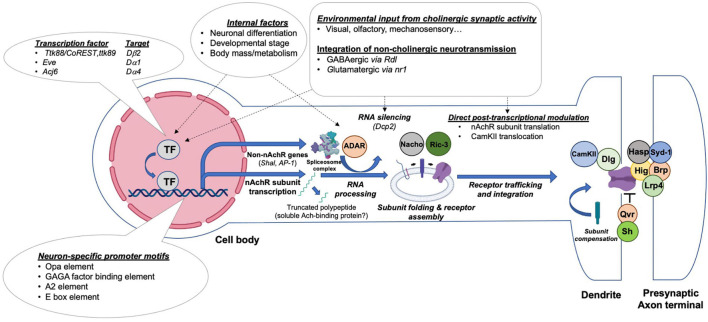
Transcriptional and post-transcriptional mechanisms regulating nAchRs’ expression, maturation, synaptic integration and activity. *Drosophila* central neurons control the production and activity of nAchRs through distinct steps by integrating regulatory influences exerted by presynaptic activity or the internal state of the animal. A simplified illustration of potential events and current molecular findings related to the transcriptional and posttranslational regulation of nAchRs is shown.

Although the temporal regulation of nAchR subunits is demonstrated for multiple subunits, the upstream transcription factors remain unclear. Nonetheless, certain regulators have been uncovered through various screens and phenotypic analyses, including Ttk88 and Eve, two transcriptional repressors, and Acj6, a transcription factor which binds to the *Dα4* and *ChAT* promoters (Estacio-Gomez et al., 2020). The *Ttk88* consensus binding site AGGG^C^/_T_GG was identified in the *Dβ2* gene, as well as several other neural-specific genes, including *para* and *synapsin* (Dallman et al., 2004). This observation, together with S2 cell transfection experiments, indicates that Ttk88 represses *Dβ2* transcription in order to inhibit neuronal differentiation in non-neural lineages. A similar phenomenon was found in aCC/RP2 motoneurons, which receive cholinergic input through nAchRs. Here, overexpression of *Eve* diminishes the strength of mEPSCs and action-potential dependent currents (Pym et al., 2006). A transcriptomic analysis revealed an *in vivo* interaction between the *Dα1* promoter and Eve. An additional overexpression experiment showed that ectopic *Eve* is sufficient to reduce the *Dα1* RNA level by almost three fold. Thus, both Eve and TTk88 function in establishing non-neuronal properties by repressing the expression of nAchR subunits (Figure 4).

### Post-translational Regulation of Nicotinic Acetylcholine Receptor Assembly and Activity by Accessory Proteins

Between the initial steps of nAchR subunit translation to the assembly of a fully operational pentameric channel at the synaptic site, multiple tightly controlled processes occur (Figure 4). Post-translational regulatory steps affect protein folding and modification, receptor assembly and trafficking, as well as their synaptic integration. Although it appears that at least several of the proteins regulating nAchR synthesis and processing are conserved between flies, worms and vertebrates, much remains to be discovered (Jones et al., 2010).

One of the early points of post-translational regulation occurs within the Golgi and ER complex, where critical molecular chaperones, such as *Ric-3*, are needed for nAchR subunit assembly. *Ric-3* was initially identified by genetic screens in *C. elegans* and found to have a conserved function in mammals and *Drosophila* (Halevi et al., 2002, 2003). In cell culture systems, *Dα2* and Rat β*2* transfection only produce epibatidine binding sites when co-transfected with d*Ric-3* and the degree of binding varies significantly between the alternatively spliced isoforms of d*Ric-3* (Lansdell et al., 2008). Dα5-Dα6 heteromers have also been produced when assisted by either d*Ric-3* or *C. elegans Ric-3* (Lansdell et al., 2012). Importantly, these artificially generated nAchRs have α-Btx binding sites and are functional at the plasma membrane, demonstrated by the production of strong Ach-gated inward currents, suggesting that Ric-3 facilitates the formation of properly folded, mature nAchR receptors. Interestingly, human *Ric-3* also facilitates the assembly of epibatidine-sensitive *Drosophila* receptors, although the efficiency varies based on the host cell type. Additional coprecipitation experiments revealed that dRic-3 physically interacts with several fly α and β subunits and even the human α7 subunit, supporting the direct chaperone activity of dRic-3 on multiple subunits.

After receptor assembly and trafficking, nAchRs rely on extracellular matrix and transmembrane proteins to ensure a stable integration to the synaptic sites. Genetic and biochemical experiments have shown that Hasp and Hig are two such factors that interact sequentially with nAchRs in the developing fly brain (Nakayama et al., 2014, 2016). In the early stages of synaptic development, the CCP domain-containing protein Hasp is secreted then localizes to cholinergic synapses. Later, the intermediate nAchR recruiter Hig, another secreted factor, is captured by Hasp. Interestingly, while the MB calyx of either *hig*- or *hasp*-deficient animals have reduced levels of synaptic Dα6 and Dα7, nAchR subunit deficiency can also cause reduced synaptic accumulation of Hig, whereas Hasp is unaffected due to its earlier presence at the synapse. In addition, the presynaptic membrane protein Lrp4 regulates excitatory cholinergic synapse number and active zone structure (Mosca et al., 2017). Interestingly, the vertebrate Lrp4 homolog is also a central component of synaptogenesis at the NMJ but is localized postsynaptically (Zhang et al., 2008).

Even after the nAchR has been stably inserted into the membrane, its activity can still be altered. For instance, to dampen nAchR-mediated excitatory neurotransmission during resting/sleep periods, the GPI-anchored protein Quiver/Sleepless (Qvr) is required for the physiological downregulation of Dα3 activity (Dean et al., 2011; Wu et al., 2014). Due to the deficit in nAchR downregulation, *qvr* mutants are characterized by a significant reduction in sleep, which can be rescued by application of the nAchR antagonist mecamylamine or knockdown of either *Dβ3* or *Dα3*, the latter of which also coprecipitates with Qvr *in vitro*. These results, together with the finding that *Dα3* and *Dβ3* RNA levels are unaltered in *qvr* mutants, support the idea of Qvr functioning as an activity modulator of fully assembled nAchRs. Finally, although phosphorylation events of *Drosophila* nAchRs have not been studied in detail, multiple subunits contain predicted phosphorylation sites within their large TM3-TM4 loop, which has been linked to receptor desensitization, and nAchRs in the vertebrate system including α4 and α7 are known targets of PKA and PKC (Gundelfinger and Hess, 1992; Broughton et al., 1996; Schulz et al., 1998; Kabbani et al., 2013).

As mentioned in previous sections, plasticity is a widespread occurrence at cholinergic synapses, and in some cases, is strongly tied to post-translational events (Figure 4). A great example is related to the case we discussed earlier: inhibition of nAchR-mediated cholinergic activity which drives the transcription-independent increase in Dα7-mediated currents, (Ping and Tsunoda, 2011; Eadaim et al., 2020). The observation that overexpressing the nAchR-associated chaperone NACHO alone can enhance this homeostatic response indicates that post-translational processes can also elevate cholinergic transmission even when total nAchR protein levels remain constant.

In summary, compared to the general expression patterns and temporal dynamics of nAchRs in the *Drosophila* CNS, much less is known about the regulatory networks that dictate the transcriptional and post-translational regulation of individual subunits. But in these areas lie great opportunities for exciting discoveries. First, while there appears to be a correlation between nAchR expression and neuronal differentiation and synaptogenesis, the upstream factors controlling this process are likely subunit- and developmental stage-specific (Rosenthal et al., 2021). Such an example can be seen in the vertebrate system: the presence of NMDA-type iGluRs at so-called “silent” synapses, whose activation requires stronger depolarization events, often temporally precedes the synaptic recruitment and integration of AMPA-type iGluRs (Malenka and Bear, 2004). Development of the cholinergic NMJ in vertebrates is also influenced by synaptic activity and is under transcriptional control. During NMJ formation, synaptic transmission is important for preventing extrasynaptic nAchR clustering. In addition, a neonatal switch in nAchR receptor composition, in which the γ subunit is replaced by the related ε subunit, has also been identified (Sanes and Lichtman, 1999, 2001). Studies on the transcription factors regulating nAchR subunits expression could lead to better understanding of common principles regulating neuronal plasticity. Secondly, nAchR abundance and activity at the synapse is clearly under tight regulation through post-translational events, where key accessory molecules contribute to the unique spatiotemporal expression patterns of their target subunits. One interesting question is how these auxiliary proteins act during activity-induced plasticity to generate acute changes in nAchR functionality, similar to the case in which NMDA receptor-dependent Ca^2+^ ion influx triggers the translocation of vesicle-bound AMPA receptors to the PSD (Park, 2018). Finally, studying various nAchR-related accessory proteins in *Drosophila* may also be an effective avenue for developing cholinergic signaling-related therapeutics. In contrast to the traditional use of nicotinic-type agonists and antagonists to directly manipulate nAchR activity, targeting various chaperones and accessory proteins might offer additional options for pharmaceutical development.

## New Strategies to Investigate the Functions and Regulation of *Drosophila* Nicotinic Acetylcholine Receptors

Since the first *Drosophila* nAchR gene was cloned over 30 years ago, the field has made tremendous headway in cataloguing the diversity of nAchR subunits and their individual functions. The traditional means, such as heterologous expression and genotype-phenotype analyses, have thus far been informative. However, new techniques have been gaining traction in the past decade and greatly complement these traditional approaches, which still have not been able to clarify native receptor subunit composition in the fly CNS. This section will allude to several of these developments which have already shown promising results since their implementation.

### Examining the Native Expression and Localization of Nicotinic Acetylcholine Receptor Subunits Using Endogenous Tagging Approach

Perhaps the most significant hurdle to understand the functions of nAchR subunits in *Drosophila* is the enduring difficulty of identifying the endogenous distribution and organization of the pentameric channels (Dupuis et al., 2012). One initial step to circumvent this barrier is to study the native expression of nAchRs *in vivo* using the endogenous tagging approach. The MiMIC (Minos-mediated integration cassette) technique developed by the Gene Disruption Project allows the insertion of either an in-frame GFP tag or a Gal4 element into the coding introns of specific subunits. Currently, more than half of the fly nAchR gene family has established MiMIC lines available from public sources, revealing the expression patterns of individual nAchR subunits (Venken et al., 2011; Gnerer et al., 2015) (Gene Disruption Project).

More recently, significant efforts have also been made to perform endogenous tagging of all neurotransmitter receptor genes using CRISPR-Cas9-mediated genomic editing. This led to a collection of T2A-Gal4 lines that contains Gal4 elements directly inserted into the C-terminus region of individual subunits, without interfering with their coding sequences (Deng et al., 2019; Kondo et al., 2020). Although currently these lines only report the subunits’ general tissue distribution, Gal4-to-GFP conversion using the RMCE (Recombination-mediated cassette exchange) technique could also create a knock-in GFP tag at the same location and reveal the native expression pattern of the subunits with subcellular resolution.

**FIGURE 5:**
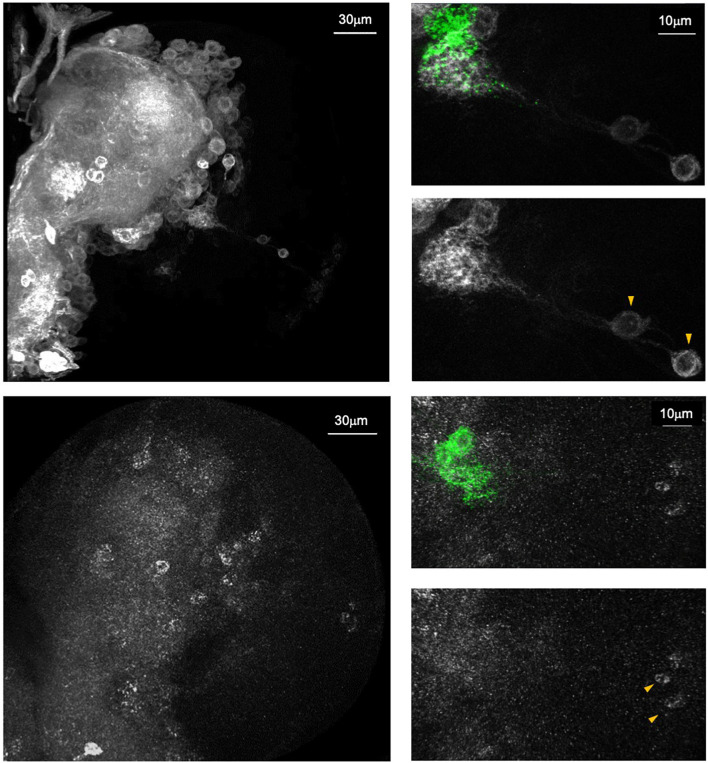
Endogenous *Dα6* expression pattern is revealed by endogenous tagging approach. **Top:** A knock-in Trojan-Gal4 gene trap in the *Dα6* locus driving mCD8::GFP (white) expression. **Bottom:** anti-HA antibody staining on a CRISPR/Cas9 engineered *Dα6::HA* allele (white). Both methods reveal similar staining profiles in the third instar brain lobe. Right panels: Magnified views of brain regions proximal to the ventral lateral neurons (LNvs)(green). Both samples show the positive labeling of the larval optic lobe pioneer neurons (IOLPs, arrowhead) (Taken from Rosenthal et al., 2021).

Both approaches mentioned above have been used successfully to demonstrate subunit-specific expression patterns of nAchRs. For example, analysis of Gal4-expressing MiMIC lines revealed that *Dα6*, but not *Dα1*, is transcriptionally active in the local optic lobe pioneer (lOLP) neurons of the larval visual circuit. This is supported by the staining pattern seen in a *Dα6*, but not *Dα1*, allele expressing an endogenously tagged receptor. Similar comparisons made between the subunit-specific T2A-Gal4 alleles were also helpful in unveiling cell-to-cell variations in expression, such as the intense signal for *Dα3*, but not *Dβ2*, in the larval LNvs (Figure 5) (Rosenthal et al., 2021). These tools have the advantage of labeling the neuropil or nuclei of the nAchR-expressing cells, thereby achieving a resolution that can be difficult to accomplish with antibody staining alone. Finally, although the knock-in T2A-Gal4 vs. MiMIC-Trojan-Gal4 staining patterns are not necessarily identical, they display similar anatomical profiles and likely reflect true expression of the subunits. This can be seen, for example, in our recent publication where both *Dα6* Gal4 lines broadly label the central neuropil and cortex layer, consistent with the endogenously tagged Dα6 protein expression.

### Genetically Encoded Sensors to Evaluate the Physiological Functions of Nicotinic Acetylcholine Receptors

As discussed in the previous sections, physiological characterization of nAchR-mediated currents and calcium flux through electrophysiological recordings has been significantly hampered by the difficulties associated with heterologous insect nAchR expression in an *in vitro* setting. To study biologically relevant nAchR channel physiology, several technologies now allow for direct *in vivo* recording from live animals and/or live tissue explants. The most widely used is calcium imaging using GCaMP, which fuses one segment of the Ca^2+^-binding protein Calmodulin with the fluorescent reporter GFP and reflects intracellular calcium concentration by changes in intensity. Different versions of calcium indicators, such as GCaMP, RCaMP and Chameleon, have been used for over 15 years in *Drosophila*, including in multiple cell types receiving cholinergic input through nAchRs (Raccuglia and Mueller, 2014; Wu et al., 2014; Barnstedt et al., 2016; Cervantes-Sandoval et al., 2017; Sheng et al., 2018; Simpson and Looger, 2018; Yin et al., 2018).

The inherent caveat of calcium imaging is that measurements of only Ca^2+^ flux may not fully reflect changes in current or membrane potential. This can be addressed by using voltage indicators. Commonly used variants, such as ASAP and ArcLight, are constructed from the voltage-sensing domain (VSD) of a tunicate and chicken voltage-sensitive phosphatase, respectively, together with GFP, and have also been efficaciously demonstrated in visual and olfactory circuits of *Drosophila* (Cao et al., 2013; Yang et al., 2016). Lastly, there is the recently developed GRAB_Ach_ (GPCR-activation-based Ach) sensor, which consists of a muscarinic Ach receptor (mAchR) and an internally placed GFP. Here, Ach binding induces the conformational change which results in fluorescence. With the sufficient sensitivity and fast response, this new type of Ach sensor has been able to shed light on the release kinetics and diffusion patterns of Ach in the mushroom bodies and antennal lobes of the adult *Drosophila* olfactory circuit (Jing et al., 2018, 2020). By identifying specific sites of Ach release, this technique may also assist in understanding whether nAchR activation in the fly CNS occurs outside the postsynaptic density at appreciable rates, as it does in the vertebrate system.

In summary, genetically encoded calcium sensors and voltage indicators have been exceptionally helpful for assessing the physiological responses evoked by cholinergic neurotransmission through live imaging. Not only do these optic recordings bypass the need for heterologous expression, they also preserve the native synaptic environment and allow for simultaneous observations of multiple cellular compartments or neuronal populations. In addition, the Ach sensors have the potential to provide the much-needed spatial resolution in order to answer questions about the activation patterns of *Drosophila* nAchRs at the subcellular level.

### Inferring Nicotinic Acetylcholine Receptor Molecular Function Through Homology Modeling

The lack of a *Drosophila* nAchR X-ray crystal structure is another area where the fly model is currently at a disadvantage. Crystallography first resolved the structure of the acetylcholine binding protein (AchBP), which is homologous to the nAchR extracellular domain, for the snail *L. stagnalis* in 2001 and later in the sea slug *A. Californica* (Brejc et al., 2001; Ulens et al., 2006). Examinations in vertebrates, using either the full protein or extracellular domain, led to the acquisition of crystal structures for multiple vertebrate subunits, including mouse α1 as well as human α2 α4, α9, and β2 (Dellisanti et al., 2007; Zouridakis et al., 2014; Kouvatsos et al., 2016; Morales-Perez et al., 2016). Structural analyses of nAchRs tremendously helped define and/or support the predictions made regarding the receptor topology, the interaction interface with various agonists/antagonists, as well as key residues mediating these interactions. Fortunately, because nAchR orthologs are generally well-conserved in amino sequence, it is feasible to model *Drosophila* subunits and theoretical receptor subtypes using their vertebrate counterparts as templates. In one example, mouse α4 was used as a template to simulate the structure of fly α1 and β2 subunits, and their interface in a pentameric channel (Liu et al., 2010). This model predicted a rapidly stabilizing complex and the free energy comparisons between the template and the model were also able to accurately predict the higher affinity of the *Drosophila* α subunit toward the insecticide imidacloprid than that of the mouse α subunit. These predications are consistent with the sensitivity disparities observed *in vivo* as well as *in vitro*. Therefore, until the *Drosophila* nAchR crystal structure is determined experimentally, the homology modeling approach could potentially be used to ascertain the general structure of fly nAchRs, and to deduce the contribution of individual amino acids to ligand affinity, ion species conductance and other biophysical/biochemical properties.

### Contextualizing Individual Nicotinic Acetylcholine Receptor Subunit Function With Global Transcriptomic and Proteomic Analyses

One technology now routinely used to comprehend gene expression at a high-throughput level, and which also holds great potential for nAchR studies, is the transcriptomics approach. This group includes a variety of techniques, such as RNA sequencing (RNA-seq) and microarrays, which have been used to in the past to interrogate distinct cell and tissue populations in *Drosophila* at various developmental stages or under different environmental conditions, such as cold exposure (Zhang et al., 2011; Karaiskos et al., 2017; Hung et al., 2020). In particular, the powerful tissue-specific bulk RNA-seq or single-cell RNA-seq (scRNA-seq) both help define the array of nAchR subunits produced in a specific cell type. For instance, cell-specific RNA-seq using adult MBs showed that V2 mushroom body output neurons (MBONs) express *Dα3*, *Dα4*, and *Dα7*, whereas the γ-type intrinsic Kenyon cells expressed *Dα3* and *Dα7*, but not *Dα4* (Crocker et al., 2016). Cell-type specific expression of nAchR subunits, as well as correlating expression between subsets of nAchR genes, are also supported by studies analyzing the mushroom bodies and other olfactory circuit elements using drop-seq and TAPIN-seq (Croset et al., 2018; Shih et al., 2019).

In recent years, new proteomics techniques have been developed to analyze the protein composition at the synaptic sites. One such example is the chemical-genetics approach, proximity labeling, which could unravel the protein-protein interactions that take place during the course of nAchR maturation and synaptic integration. As mentioned earlier in this review, co-immunoprecipitation (Co-IP) experiments have indicated potential permutations of subunit co-assembly, which can be further corroborated by scRNA-seq datasets. However, co-IP lacks the cellular resolution and could introduce artifacts due to the non-native environment and altered protein concentrations introduced by the sample preparation. These limitations can be addressed partly through proximity labeling, which not only entails cell-specific labeling, but also reflects the spatial proximity within a small radius (i.e., several nanometers), and therefore is ideal to capture the dynamic interactions between nAchR subunits and their accessory proteins in different neuronal types. This nascent technology has already been tested and validated in *Drosophila* and expanded the known components of protein-protein networks relating to ring canal (RC) bridges functioning during gametogenesis as well as the Ecdysone receptor/Ultraspiracle (EcR/USP) transcriptional regulator complex (Mannix et al., 2019; Mazina et al., 2020). Given its successful applications in mammalian synapses (Branon et al., 2018; Cho et al., 2020), proximity labeling may be employed to determine both the native compositions of pentameric nAchR channels, as well as the accessory proteins that facilitate each nAchR subunit’s synaptic localization and function.

## Concluding Remarks

The structure, function and regulation of nAchRs has been a premier research topic in neuroscience, a trend that will likely persist given its multifaceted roles in nervous systems across the animal kingdom. Toward this end, we consider excitatory cholinergic transmission in *Drosophila* CNS as an effective model to study nAchR-mediated signaling. Not only does it serve the purpose for expediting molecular discoveries related to central cholinergic synapse development and plasticity, but it also has relevance for insect-specific questions, such as modeling insecticide resistance in wild populations, and understanding the species-specific usage of nAchR subunits for a wide variety of behaviors and cognitive processes. And as technologies advance, the field will move closer to solving longstanding questions, including the compositions of endogenous fly nAchR pentamers, as well as how these receptors are globally regulated by transcriptional and post-transcriptional mechanisms to achieve specific distributions and functions in a time- and context-dependent fashion.

## Author Contributions

JR and QY wrote the manuscript. JR generated the schematic diagrams. Both authors contributed to the article and approved the submitted version.

## Conflict of Interest

The authors declare that the research was conducted in the absence of any commercial or financial relationships that could be construed as a potential conflict of interest.

## Publisher’s Note

All claims expressed in this article are solely those of the authors and do not necessarily represent those of their affiliated organizations, or those of the publisher, the editors and the reviewers. Any product that may be evaluated in this article, or claim that may be made by its manufacturer, is not guaranteed or endorsed by the publisher.
